# Examining family processes linked to adolescent problem behaviors in single-mother families: The moderating role of school connectedness

**DOI:** 10.3389/fpsyg.2022.937698

**Published:** 2022-09-26

**Authors:** Woon Kyung Lee, Young Sun Joo

**Affiliations:** ^1^Department of Child Development and Intervention, Ewha Womans University, Seoul, South Korea; ^2^School of Social Welfare, Myongji University, Seoul, South Korea

**Keywords:** single mother, adolescent, internalizing problems, externalizing problems, school connectedness

## Abstract

**Objective:**

Previous research has shown that adolescents in single-mother households are at heightened risk for adjustment problems. However, limited studies have investigated the mechanisms leading to adolescent problem behaviors in single-mother households. To address this research gap, this study applied the Family Stress Model to examine how single mothers’ material hardship is linked to adolescent problem behaviors, focusing on the mediating roles of mothers’ depression and mother-adolescent closeness. The moderating role of adolescent school connectedness in the relationships between mother-adolescent closeness and school connectedness and between mothers’ depression and school connectedness was also investigated.

**Materials and methods:**

The study analyzed data from 1,384 adolescents and their single mothers who participated in the Fragile Families and Child Wellbeing Study at Wave 6. The associations between study variables were analyzed using structural equation modeling by decomposing the direct, indirect, and total effects of material hardship on adolescent problem behaviors. School connectedness’s interactions with mother-adolescent closeness and mothers’ depression were also examined.

**Results:**

Results showed a significant indirect relationship between material hardship and adolescent problem behaviors through sequential mediation of mothers’ depression and mother-adolescent closeness. Mothers’ depression also significantly mediated the relationship between material hardship and problem behaviors. Lastly, school connectedness moderated the relationship between mother-adolescent closeness and adolescents’ internalizing behaviors. The association between mother-adolescent closeness and adolescents’ internalizing behavior was weaker for adolescents with higher levels of school connectedness.

**Conclusion:**

The results indicate the important indirect effect of economic strain on adolescents’ problems behaviors in single-mother households, which has been less emphasized compared to the effects in earlier childhood. High rates of material hardship and adolescent problem behaviors in single-mother families call for multifaceted interventions focusing on family processes and protective factors, including school environment.

## Introduction

The number of single-parent families with children under the age of 18 has increased sharply during the past few decades in the United States. There were approximately 10.5 million single-parent households in 2021, compared to 1.5 million in 1950 (United States Census Bureau, 2022). A recent study indicates that among countries where census data are available, the rate of single-parent households is highest in the United States (Pew Research Center, 2019). As the number of single-parent families has increased, many studies have examined children’s and adolescents’ developmental outcomes in single-parent (especially single-mother) families. For example, multiple studies indicate that adolescents living in single-mother households are at heightened risk for adjustment problems, including internalizing issues—difficulties directed primarily inward (Loeber and Burke, 2011)—such as depression and anxiety (Barrett and Turner, 2005; Amato, 2010; Turner et al., 2013; King et al., 2018), as well as outwardly directed externalizing issues, such as earlier initiation of substance use (Donovan and Molina, 2011), greater likelihood of engaging in delinquency (Dornbusch et al., 1985; Thomas et al., 1996), and attention-deficit hyperactivity disorder and conduct disorder (Daryanani et al., 2016).

Unlike early research that reflected concerns about single-parent households, recent research indicates that the family structure itself is not the dominant influence on adolescents’ developmental outcomes (Cohen et al., 2015). Many children in single-parent families develop into well-adjusted adults (Solomon-Fears, 2014), and in general, recent studies report more multifaceted elements of single parenthood, including both resilience and risk factors, compared to earlier research (Taylor and Conger, 2017). Nevertheless, some risk factors associated with single-parent families have consistently emerged as predictors of adolescents’ problem behaviors. For instance, high rates of material hardship (Amato, 2000), single parents’ depression (Compas and Williams, 1990), and less optimal parenting behaviors (e.g., low involvement and monitoring, and harsh discipline) are key risk factors for adolescent problem behaviors in single-parent families (Laursen, 2005). Although examining the main risk factors for adolescents’ problem behaviors is very important, identifying specific mechanisms underlying family processes that include those risk factors is necessary to explain the formation and progression of adolescents’ problem behaviors (Restifo and Bögels, 2009; Childs et al., 2020). Nevertheless, few studies have explored family processes in single-parent households to understand adolescent problem behaviors (e.g., Turner et al., 1991; Taylor et al., 2010).

Specifically, the higher poverty rate of single-parent households compared to two-parent households is a major risk factor linked to children’s developmental outcomes (Weinraub and Kaufman, 2019). Conger et al. (1994) proposed the Family Stress Model (FSM), which explains adolescents’ problem behaviors through family processes that originate with parents’ economic burden. Particularly, considering that female-headed single-parent households are more likely to be impoverished compared to male-headed households and that financial problems are a major stressor in single-mother households (Weinraub and Kaufman, 2019; United States Census Bureau, 2022), applying the FSM to examine single-mother families’ processes that lead to adolescents’ problem behaviors could provide valuable insights.

This study extends previous studies that have employed the FSM, examining the potentially protective role of adolescent school connectedness in the family processes that lead to adolescent problem behaviors. School connectedness is students’ experience of belonging and engagement in school, which is an important protective factor for adolescent’s healthy development (Bond et al., 2007; Monahan et al., 2010). Family and school are two important institutions for adolescents, providing emotional and social resources for healthy development. According to ecological systems theory (Bronfenbrenner, 1989; Bronfenbrenner and Morris, 2006), interactions between the two institutions are conceptualized as a “mesosystem,” which refers to relationships among two or more immediate settings. Although studies considering the role of other institutions in investigating families’ effects on children’s and adolescents’ outcomes are scant (Parcel et al., 2010), some research has indicated the joint (i.e., not merely additive) effect of the two institutions on development. For example, one study revealed the compensating effect of school on behavioral problems for children from families with lower socioeconomic resources (Domina, 2005). Additionally, the influence of family violence on adolescents’ peer aggression is mitigated when adolescents have a stronger sense of belonging in school (Valido et al., 2021). With few previous studies examining moderating effects within the FSM, this study focuses on the moderating role of adolescents’ school connectedness in the paths to their problem behaviors to determine the impact of other important environmental factors on the development of adolescents in single-mother families.

### Applying the FSM: The indirect association between single mothers’ material hardship and adolescent problem behaviors

The FSM (Conger et al., 1994, 2010) delineates how parents’ emotional distress related to economic hardship can impact children’s developmental outcomes. The model indicates that economic pressure (e.g., inability to pay monthly bills) is not only a family’s objective condition but also a factor that affects parents and children psychologically (Conger and Donnellan, 2007). Instead of focusing on economic hardship’s direct effect on children’s adjustment, the FSM posits comprehensive family processes, such as parents’ emotional and behavioral functioning, as mediating mechanisms. Specifically, parents’ economically influenced emotional distress (e.g., depression and anxiety) can significantly disrupt their ability to maintain positive and nurturing parent–child relationships. Less adaptive behaviors such as harsh and uninvolved parenting in turn can lead to children’s emotional and behavioral problems and impaired competence (Conger et al., 2010).

Empirical studies testing the FSM have demonstrated that it is a valid heuristic model that can be applied to families with children in diverse developmental stages (e.g., Mistry et al., 2002; Parke et al., 2004; Benner and Kim, 2010). Research has indirectly linked parents’ experience of economic hardship to behavioral problems of children in early and middle childhood *via* parents’ emotional distress, couples’ conflicts, and harsh parenting behaviors (Linver et al., 2002; Solantaus et al., 2004; Neppl et al., 2016; Sosu and Schmidt, 2017). Studies targeting families of adolescent children with reports from both mothers and fathers (regardless of family structure) have also demonstrated the significant indirect effect of economic hardship on adolescents’ behavioral problems through family processes (Benner and Kim, 2010; Ponnet, 2014; Landers-Potts et al., 2015; Ponnet et al., 2016; Diggs and Neppl, 2018). Empirical evidence for the FSM’s validity has also been established across diverse family contextual backgrounds. For example, economic pressure’s indirect effects on internalizing and externalizing problems have been reported in samples with various ethnic backgrounds, such as families of African, Chinese, and Mexican origin, as well as in samples with diverse geographic backgrounds (Benner and Kim, 2010; Ponnet, 2014; Landers-Potts et al., 2015; Sun et al., 2015; White et al., 2015; Ponnet et al., 2016; Simons et al., 2016).

However, most studies applying the FSM to explain adolescent behavioral problems have targeted two-parent families or included parents regardless of their gender (Yoder and Hoyt, 2005; Wadsworth et al., 2013; Ponnet, 2014; Sun et al., 2015; Ponnet et al., 2016; Diggs and Neppl, 2018), with little focus on diverse family structures. Additionally, although some studies have explored stress processes in single-mother families, most of them targeted single mothers of younger children (Brody and Flor, 1997; Mistry et al., 2002; Scaramella et al., 2008), while fewer studies (e.g., Kim and Brody, 2005; Turner et al., 2013) have focused on identifying how family processes in single-mother households are linked to adolescents’ adjustment.

Adolescents’ healthy development is important in itself, but preventing and mitigating adolescents’ adjustment problems is significant, as such problems can affect individuals throughout adulthood. Although few empirical studies have assessed how single mothers’ economic hardship is linked to adolescent problem behaviors *via* mental health and parent-adolescent relationships, previous studies that have examined parts of the FSM support the viability of the current study’s research model. Previous research indicates that on average, single mothers tend to suffer from higher levels of stress compared to married mothers (Cairney et al., 2003). Specifically, financial problems are the main stressor for single mothers and significantly impede their psychological functioning (Kim and Brody, 2005; Kotwal and Prabhaker, 2009). For example, studies have showed that single mothers’ financial hardship is associated with their current and chronic depressive symptoms (Brody and Flor, 1997; Brown and Moran, 1997; Kim et al., 2018).

Several studies have also reported the mediating role of parenting behaviors or parent-adolescent relationships in the relationship between mothers’ mental health problems and adolescents’ behavioral problems (e.g., Cummings and Davis, 1994; Johnson and Greenberg, 2013), although they did not focus on single mothers. Single-mother status itself may not be associated with insufficient functioning as parents (Turner et al., 1991), but single mothers’ emotional difficulties (e.g., depression and anxiety) are associated with less optimal parenting behaviors, such as low levels of authoritative and involved parenting, punishment, physical abuse, and neglect (Eamon and Zuehl, 2001; Leinonen et al., 2002). In addition, the quality of parenting behaviors and parent-adolescent relationships have consistently been found to be strong predictors of adolescent problem behaviors (Soenens et al., 2006; Johnson and Greenberg, 2013). Specifically, instead of focusing on the disruptive parenting behaviors indicated as mediators between parents’ and children’s maladjustment in FSM, this study focused on the mediating role of the mother-adolescent closeness between single mothers’ depression and adolescents’ problem behaviors. The reason for this is that the bond between a parent and an adolescent (e.g., closeness, connectedness, attachment) can be an essential facet of the relationship that explains adolescents’ adjustment (Clark and Ladd, 2000; Collins and Laursen, 2004; Pinquart, 2014). The quality of parenting behaviors and parent-adolescent relationships have consistently been found to be strong predictors of adolescent problem behaviors (Soenens et al., 2006; Johnson and Greenberg, 2013). Specifically, the closeness between a parent and an adolescent is an important relationship factor that is often measured by indicators such as trust, intimacy, and communication (Collins and Laursen, 2004; Branje et al., 2012). Closeness between a parent and an adolescent is often measured by indicators such as trust, intimacy, and communication (Collins and Laursen, 2004; Branje et al., 2012). Lower levels of mother-adolescent closeness, mother-adolescent communication, affection, and care are associated with higher levels of externalizing and internalizing problems among adolescents (Barrett and Turner, 2005; King et al., 2018; Coates et al., 2019). Adolescents’ weak sense of belonging and low levels of trust and attentive listening between mothers and adolescents are mechanisms explaining the link between closeness with parents and problem behaviors (Smetana et al., 2002; King et al., 2018).

In addition to the possibility that single mothers’ economic hardship is linked to adolescent problem behaviors through mothers’ mental health and the mother-adolescent relationship, a few studies also indicate that mothers’ depression may be directly associated with adolescent problem behaviors, even after accounting for the mediating role of parent-adolescent relationships. Emotional contagion among family members and genetic vulnerability to depression are a few mechanisms potentially explaining parents’ mental health and children’s negative adjustment (Goodman and Gotlib, 1999; Wolford et al., 2019).

### Adolescent school connectedness as a moderator

There have been few studies examining potential risk or protective factors that moderate family stress processes (Masarik and Conger, 2017). Previous studies have indicated that the level of poverty (Ponnet, 2014), mothers’ personality and values (Taylor et al., 2010; Gonzales et al., 2014; White et al., 2015), and coping strategies and social support (Wadsworth et al., 2013; Taylor et al., 2014) play significant moderating roles, exacerbating or mitigating stress processes. Specifically, in studies examining how stress processes are linked to problem behaviors, the moderating role of neighborhood characteristics was reported, including collective efficacy in the relationship between harsh parenting and behavioral difficulties (Krishnakumar et al., 2014), as well as neighborhood adversity in the relationship between harsh parenting and adolescents’ internalizing problems (White et al., 2015).

Although school is a significant institution in which adolescents spend most of their time outside of family, providing multiple resources for resilience development (Masten, 2014), adolescents’ relationship with school has seldom been examined in studies applying the FSM. Research shows that adolescents report better well-being when they feel like part of their school and are cared for by people in school (Eccles et al., 1997). There is also a wealth of studies reporting the relationship between stronger school connectedness and reduced externalizing and internalizing problems (Gonzales et al., 2014; Marraccini and Brier, 2017; Olivier et al., 2020). Adolescents’ superior behavioral outcomes are explained by diverse resources in schools such as the opportunity to develop secure relationships that are the basis for social and emotional development, as well chances to build psychological skills in a warm and supportive atmosphere, observing positive adult and peer role models, learning important values in life, and participating in intervention programs aimed at enhancing socioemotional development (Catalano et al., 2004; Masten and Cicchetti, 2016). Many studies have examined the effect of school connectedness on adolescents’ problem behaviors, focusing on variables related to school context (Wilson, 2004; Loukas and Pasch, 2013), with several reporting that both positive parent characteristics and school connectedness significantly decrease adolescent problem behaviors (Mrug and Windle, 2009; Duggins et al., 2016). Considering that few studies have tested the moderating effect of school connectedness in the relationship between parent characteristics/parent-adolescent relationship characteristics and problem behaviors (Loukas et al., 2010; Tian et al., 2019), this study focused on the moderating role of school connectedness in the relationships between mothers’ depression and adolescent problem behaviors as well as between mother-adolescent closeness and adolescent problem behaviors. Material hardship can negatively impact mothers’ depression and mother-adolescent closeness, but the buffering hypothesis posits that a strong social support system mitigates the negative effects of stressful events (Cohen and Wills, 1985; Landers-Potts et al., 2015; Berry et al., 2016). In short, previous studies indicate that more studies are needed to understand how parental characteristics and adolescent school connectedness may interact to influence adolescents’ adjustment in diverse contexts.

### The present study

**Figure 1:**
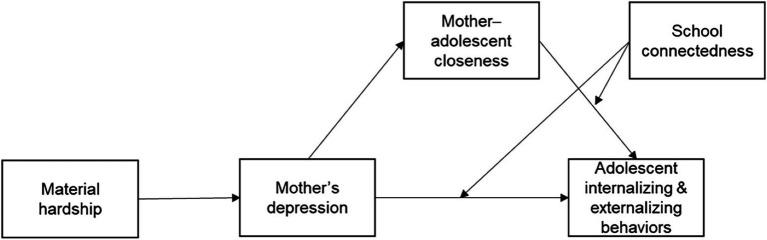
Research model.

Based on the FSM and the findings from previous research, we hypothesize that (1) single mothers’ material hardship is indirectly associated with adolescents’ internalizing and externalizing problem behaviors through sequential mediations of mothers’ depression and mother-adolescent closeness, (2) single mothers’ material hardship is indirectly associated with adolescent internalizing and externalizing problem behaviors through mothers’ depression, and (3) school connectedness moderates the association between mother-adolescent closeness and adolescent problem behaviors and the association between mothers’ depression and adolescent problem behaviors. Figure 1 shows our research model.

## Materials and methods

### Data and sample

This study analyzed data from the Fragile Families and Child Wellbeing Study (FFCWS), a longitudinal study that follows children born in the US between 1998 and 2000. The original sample was a stratified random sample of 4,898 children born in 20 cities (Wave 1) that was nationally representative, and these individuals were followed beginning at ages one (Wave 2), three (Wave 3), five (Wave 4), nine (Wave 5), and 15 (Wave 6), approximately. We analyzed data only from Wave 6 (when individuals were about age 15). The FFCWS includes both children and their primary caregivers’ interviews regarding their cognitive and behavioral development, health, parenting behaviors, neighborhoods, and other important life variables. We restricted the analysis to single mothers whose primary caregiver (PCG) report of the relationship to the adolescent was biological, representing 1,384 adolescents and their mothers. We excluded cohabiting mothers because single mothers living alone are more likely to face economic difficulties.

Table 1 displays the descriptive characteristics of the analyzed sample. Among the sample’s adolescents, 50% were female, and 9% were White, 58% were Black, 17% were Hispanic, and 6% reported the “other” racial category. Adolescents’ average age was 15.63 years (*SD* = 0.76). For mothers, the average age was 40.48 years (*SD* = 6.01). Approximately 14% of mothers had graduated from college, and 44% were living in poverty.

**Table 1:** Summary statistics (*N* = 1,384).

Variables	*M* / %	*SD*	Min	Max
Internalizing behavior	2.11	2.52	0	15
Externalizing behavior	4.97	5.39	0	36
Mother-adolescent closeness	2.26	0.77	0	3
Mothers’ depression	1.35	2.44	0	8
Material hardship	1.61	1.92	0	10
School connectedness	2.38	0.61	0	3
**Covariates**				
Adolescent is female	50%			
Adolescents’ age	15.63	0.76	14	19
Adolescents’ race				
White	9%			
Black	58%			
Hispanic	17%			
Other race	6%			
Missing	10%			
Mothers’ age	40.48	6.01	30	63
Mother graduated from college	14%			
Missing mother graduated from college	1%			
Family poverty status	44%			
Missing family poverty status	0%			

### Measures

#### Internalizing and externalizing behaviors

Adolescent behavioral development was assessed using self-report questionnaires based on the 34 items of the behavioral, emotional, and social problems scales of the Child Behavior Checklists (CBCL)/6–18 (Achenbach and Rescorla, 2001). The subscales included were aggressive behavior, anxious/depressed behavior, attention problems, rule-breaking behavior, social problems, thought problems, and withdrawal.

We calculated a score of internalizing behaviors by summing responses to six items regarding anxious/depressed behavior and two items regarding withdrawal, each measured on a 3-point Likert scale: 0 (not true), 1 (sometimes true), or 2 (often true). The composite score ranged from 0 to 15. For externalizing behaviors, we summed responses to 11 items regarding aggressive behaviors and nine items in rule-breaking behaviors, each measured on a 3-point Likert scale: 0 (not true), 1 (sometimes true), or 2 (often true). The composite score ranged from 0 to 36. The Cronbach’s alpha was.58 for the internalizing behaviors score and.89 for the externalizing behaviors score. The average scores of internalizing behaviors was 2.11 (*SD* = 2.52) and externalizing behaviors was 4.97 (*SD* = 5.39).

#### Material hardship

We assessed material hardship using the 11 items from PCG’s questionnaire, which was originally adapted from the survey on Income and Program Participation (SIPP), New York City Social Indicators Survey (SIS), and the Study of Work, Welfare, and Family Well-Being of Iowa families on FIP (Iowa’s assistance program). Each questionnaire contained yes/no questions on the extent to which respondents experienced hunger, homelessness, utility shut-offs, and forgone medical care due to insufficient financial resources. We summed responses on items to generate a composite score, with higher scores indicating greater material hardship. The scale’s Cronbach’s alpha was.74. The average score of material hardship was 1.61 (*SD* = 1.92).

#### Mothers’ depression

The major depressive episode PCG questionnaires were derived from the Composite International Diagnostic Interview-Short Form (CIDI-SF) Section A (Kessler et al., 1998), whose criteria are consistent with those of the fourth edition of the Diagnostic and Statistical Manual of Mental Disorders (DSM-IV; American Psychiatric Association, 1994). Respondents indicated whether they have had feelings of depression or a general lack of pleasure in the past year that lasted for two or more weeks. If those symptoms lasted most of the day or occurred every day within the two-week period, respondents were asked more specifically about whether they had lost interest, felt tired, experienced changes in weight, had trouble sleeping, had trouble concentrating, felt worthless, or thought about death. We then calculated the major depressive score as the sum of the positive responses to each item, which ranged from 0 to 8. The average score of mothers’ depression was 1.35 (*SD* = 2.44).

#### Mother-adolescent closeness

We assessed mother-adolescent closeness using two self-report questionnaires asking adolescents to rate the extent to which they (1) talk and (2) exchange ideas with their mothers. Each item utilized a 4-point Likert response scale ranging from 1 (extremely close) to 4 (not very close). This study recoded responses on a 0–3 scale and averaged the two items’ responses to create a composite score [Bendheim-Thoman Center for Research on Child Wellbeing (CRCW) and Columbia Population Research Center (CPRC), 2018]. The average mother-adolescent closeness score was 2.26 (*SD* = 0.77), and the scale’s Cronbach’s alpha was.49.

#### School connectedness

We assessed school connectedness using self-report questionnaires completed by adolescents, which were used in the Panel Study of Income Dynamics Child Development Supplement. The measure comprises four items assessing inclusiveness, closeness, happiness, and safety experienced at school. Items utilized a 4-point Likert response scale ranging from 1 (strongly disagree) to 4 (strongly agree), with higher scores indicating higher levels of school connectedness. We averaged responses to the items to create a composite score of school connectedness. The scale’s Cronbach’s alpha was.72 and the average score of school connectedness was 2.38 (*SD* = 0.61).

#### Covariates

This study also assessed the following adolescent and family characteristics: adolescents’ gender (male = 0, female = 1), adolescents’ age, adolescents’ race (each category dummy-coded for White, Black, Hispanic, other racial category), mothers’ age, mothers’ college graduation status (did not graduate from college = 0, graduated from college = 1), and family poverty status (family not in poverty = 0, family in poverty = 1).

### Analytic strategies

This study used path analysis, a structural equation modeling (SEM) approach, to examine the associations among material hardship, mothers’ depression, mother-adolescent closeness, and adolescents’ internalizing and externalizing behaviors. SEM considers associations among multiple pathways simultaneously and provides standardized regression coefficient for each pathway. We used bootstrapping to compute bias-corrected standard errors (MacKinnon, 2008), then we decomposed the direct, indirect, and total effects by using the nonlinear combination-of-estimators function to test for mediations. Nonlinear combination-of-estimators computes standard errors based on the delta method (Preacher and Hayes, 2008). Missing data rates for the study variables ranged from 0 to 9.68%. We used the full-information maximum likelihood estimation to retain as many observations as possible and to mitigate missing data bias. With respect to school connectedness’s moderating effect, we included interaction terms between mother-adolescent closeness and school connectedness and between mothers’ depression and school connectedness in the regression models. Then, we created a margins plot to illustrate the prediction of adolescents’ internalizing and externalizing behaviors by (1) mother-adolescent closeness and school connectedness and (2) mothers’ depression and school connectedness. We performed all analyses using Stata 14.0 (StataCorp, 2015, College Station, TX, United States).

## Results

### Correlational analysis

Table 2 shows Pearson correlations for the primary variables. Adolescents’ internalizing and externalizing behaviors, mother-adolescent closeness, mothers’ depression, material hardship, and school connectedness all were statistically significantly associated, with correlations ranging from −0.20 to.53. Adolescents’ age was associated with school connectedness, and mothers’ age was associated with adolescent externalizing behaviors, material hardship, and adolescents’ age.

**Table 2:** Correlations of primary variables (*N* = 1,384).

Correlation		1		2		3		4		5		6		7		8
		*r*	*p*	*r*	*p*	*r*	*p*	*r*	*p*	*r*	*p*	*r*	*p*	*r*	*p*	*r*
1	Internalizing behaviors	1.00														
2	Externalizing behaviors	0.53	<0.001	1.00												
3	Mother-adolescent closeness	−0.10	<0.001	−0.16	<0.001	1.00										
4	Mother’s depression	0.22	<0.001	0.21	<0.001	−0.06	0.02	1.00								
5	Material hardship	0.18	<0.001	0.21	<0.001	−0.07	0.01	0.32	<0.001	1.00						
6	School connectedness	−0.17	<0.001	−0.20	<0.001	0.22	<0.001	−0.06	0.02	−0.10	<0.001	1.00				
7	Adolescent’s age	−0.03	0.24	−0.02	0.39	0.02	0.50	0.00	0.95	−0.02	0.51	−0.06	0.05	1.00		
8	Mother’s age	0.00	0.90	−0.12	<0.001	0.00	0.89	−0.04	0.18	−0.11	<0.001	0.02	0.40	0.10	<0.001	1.00

### Associations among material hardship, mothers’ depression, mother-adolescent closeness, and adolescent internalizing and externalizing behaviors

**Figure 2:**
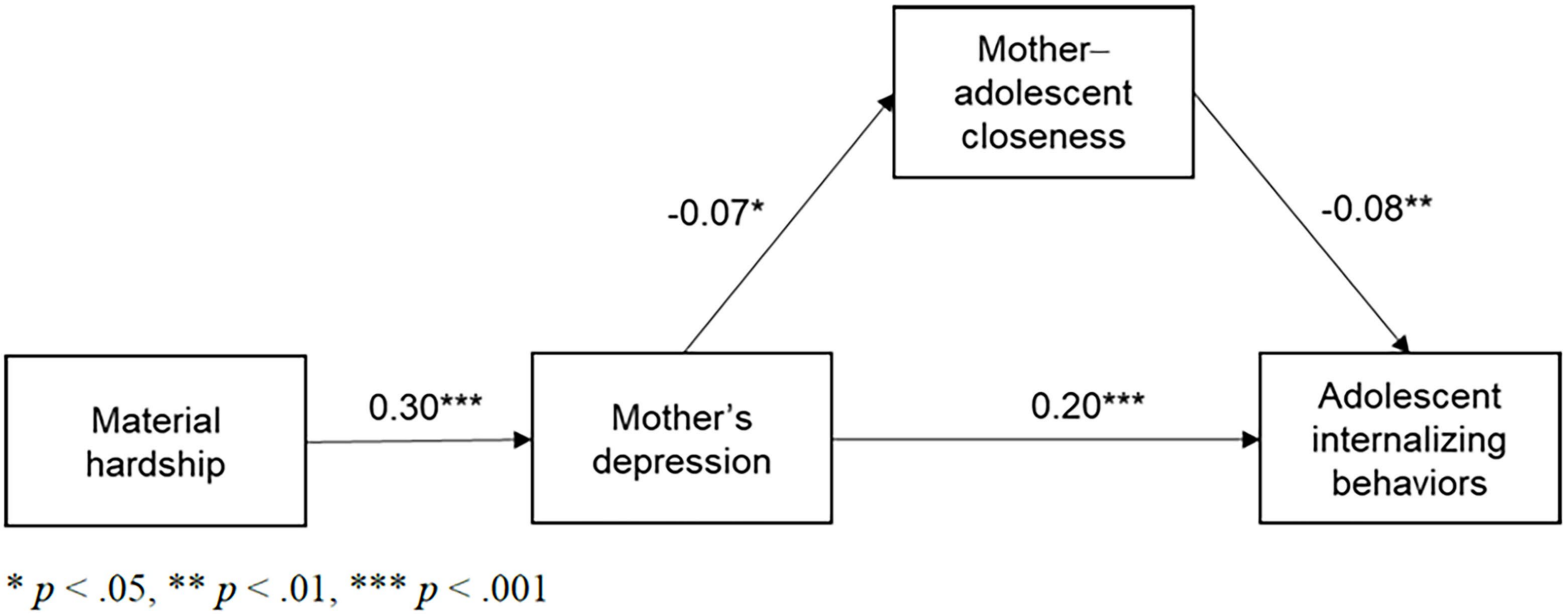
SEM path model from material hardship to adolescents’ internalizing behaviors (standardized).

All variables were observed variables and all models demonstrated good overall fit to the data. Our first SEM model predicting the path from material hardship to adolescent internalizing behavior (Table 3; Figure 2) suggested statistically significant paths *via* mothers’ depression and mother-adolescent closeness. Increases in material hardship were associated with increases in mothers’ depression (*b* = 0.30, *p* < 0.001), and increases in mothers’ depression were associated with decreases in mother-adolescent closeness (*b* = −0.07, *p* = 0.01), which in turn decreased adolescents’ internalizing behaviors (*b* = −0.08, *p* = 0.00). Mothers’ depression was directly associated with increases in adolescents’ internalizing behaviors (*b* = 0.20, *p* < 0.001).

**Table 3:** SEM Standardized coefficient estimates (*N =* 1,384).

Predictors	Direct effect											
	Mothers’ depression			Mother-adolescent closeness			Adolescents’ internalizing behaviors			Adolescents’ externalizing behaviors		
	*b*	SE	*p*	*b*	SE	*p*	*b*	SE	*p*	*b*	SE	*p*
Material hardship	0.30	(0.02)	<0.001	–			–			–		
Mothers’ depression	–			−0.07	(0.03)	0.01	0.20	(0.03)	<0.001	0.17	(0.03)	<0.001
Mother-adolescent closeness	–			–			−0.08	(0.03)	0.00	−0.16	(0.03)	<0.001
Covariates												
Adolescent is female	−0.05	(0.03)	0.05	−0.06	(0.03)	0.03	0.03	(0.03)	0.24	−0.08	(0.03)	0.00
Adolescents’ age	0.01	(0.03)	0.73	0.01	(0.03)	0.65	−0.02	(0.03)	0.42	−0.01	(0.03)	0.60
Adolescents’ race (White = 0)												
Black	−0.16	(0.04)	<0.001	0.05	(0.05)	0.30	−0.29	(0.05)	<0.001	−0.02	(0.05)	0.61
Hispanic	−0.15	(0.04)	<0.001	0.06	(0.04)	0.20	−0.19	(0.04)	<0.001	−0.10	(0.04)	0.02
Other race	−0.08	(0.03)	0.02	−0.02	(0.04)	0.51	−0.07	(0.03)	0.03	−0.04	(0.03)	0.21
Mothers’ age	−0.01	(0.03)	0.63	0.01	(0.03)	0.60	−0.02	(0.03)	0.35	−0.11	(0.03)	<0.001
Mother graduated from college	−0.01	(0.03)	0.63	−0.04	(0.03)	0.16	0.00	(0.03)	0.94	−0.02	(0.03)	0.41
Family in poverty	0.10	(0.03)	<0.001	0.03	(0.03)	0.36	0.06	(0.03)	0.03	0.08	(0.03)	0.00
Intercept	0.47	(0.56)	0.40	2.57	(0.60)	<0.001	1.97	(0.57)	0.00	2.43	(0.58)	<0.001

**Figure 3:**
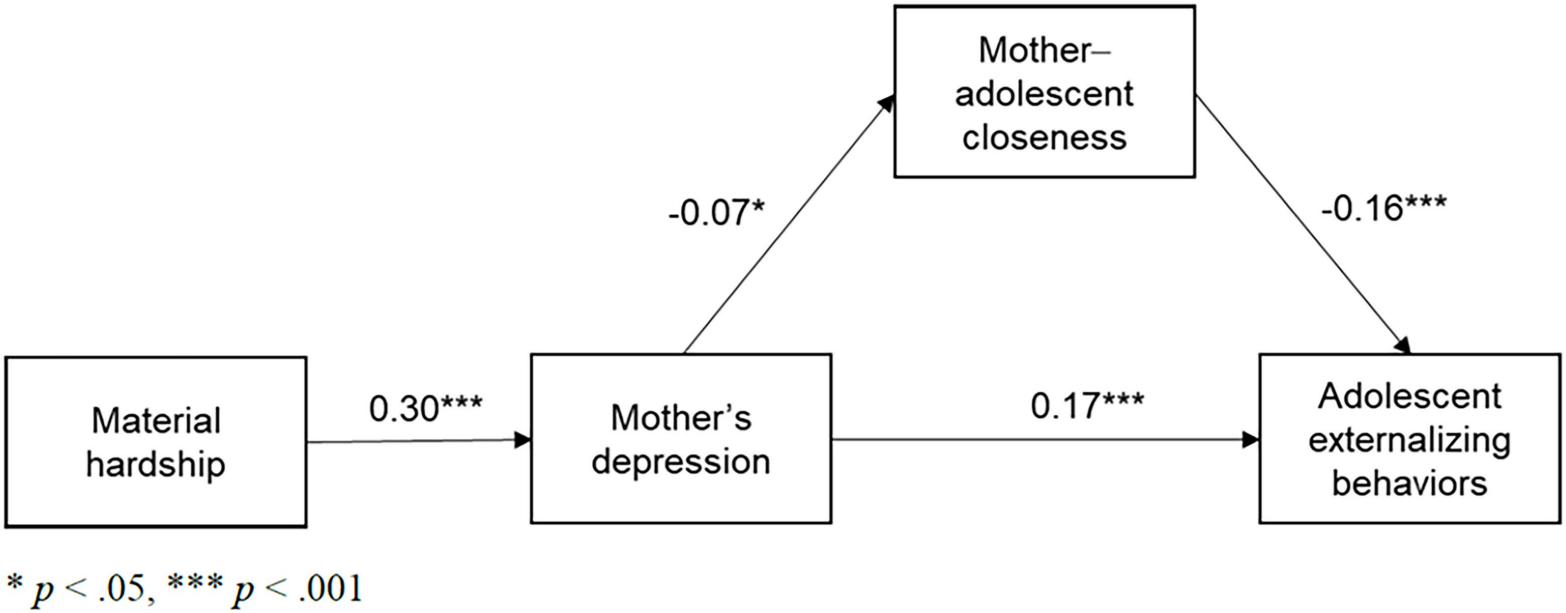
SEM path model from material hardship to adolescents’ externalizing behaviors (standardized).

The results for adolescents’ externalizing behaviors (Table 3; Figure 3) were similar to those for internalizing behaviors. Mothers’ depression was associated with increases in mother-adolescent closeness (*b* = −0.07, *p* = 0.01), which in turn decreased adolescents’ externalizing behaviors (*b* = −0.16, *p* < 0.001). Mothers’ depression was also directly associated with adolescents’ externalizing behaviors (*b* = 0.17, *p* < 0.001).

Table 4 displays the indirect effects *via* mothers’ depression and mother-adolescent closeness. Results indicated statistically significant effects for all indirect pathways. Material hardship was indirectly associated with adolescents’ internalizing (*b* = 0.08, *p* < 0.001) and externalizing (*b* = 0.17, *p* < 0.001) behaviors *via* mothers’ depression and mother-adolescent closeness. Additionally, material hardship was indirectly associated with adolescents’ internalizing (*b* = 0.08, *p* < 0.001) and externalizing (*b* = 0.14, *p* < 0.001) behaviors *via* mothers’ depression.

**Table 4:** Indirect effects (*N* = 1,384).

Model	Coef.	SE	*p*
**Hypothesis 1**			
Material hardship → mothers’ depression → closeness → internalizing behaviors	0.08	(0.01)	<0.001
Material hardship → mothers’ depression → closeness → externalizing behaviors	0.17	(0.03)	<0.001
**Hypothesis 2**			
Material hardship → mothers’ depression → internalizing behaviors	0.08	(0.01)	<0.001
Material hardship → mothers’ depression → externalizing behaviors	0.14	(0.03)	<0.001

Results are based on 200 bootstrapped estimates. All covariates are included in the analyses.

For supplementary analyses, this study tested the alternative models switching mother-adolescent closeness and mother’s depression (shown in Supplementary Table A1 and Supplementary Figures A1, A2). The results were consistent in that material hardship was indirectly associated with adolescents’ internalizing and externalizing behaviors sequentially *via* mother-adolescent closeness and mothers’ depression. This study also tested an SEM path model including both adolescents’ internalizing and externalizing behaviors within the same model since these behaviors are closely related. The findings were similar to those analyzed in separate models. In addition, the original models had smaller Akaike Information Criteria (AIC; Akaike, 1979) and Bayes Information Criteria (BIC; Raftery, 1993) values than the alternative models, indicating better model fit (Kuha, 2004) (model comparisons shown in Supplementary Tables A2, A5).

In terms of the covariates (Table 3), female adolescents had mothers who were less depressed compared to male adolescents (*b* = −0.05, *p* = 0.05), and Black adolescents (*b* = −0.16, *p* < 0.001), Hispanic adolescents (*b* = −0.15, *p* < 0.001), and adolescents reporting “other” as their race (*b* = −0.08, *p* = 0.02) had mothers who were less depressed compared to White adolescents. Living in poverty was positively associated with mothers’ depression (*b* = 0.10, *p* < 0.001). However, adolescents’ age and mothers’ age were not significantly associated with mothers’ depression. Regarding mother-adolescent closeness, female adolescents were less close with their mothers than were male adolescents (*b* = −0.06, *p* = 0.03). However, adolescents’ age, adolescents’ race, mothers’ age, mothers’ educational attainment, families’ living in poverty were not significantly associated with mother-adolescent closeness. Additionally, Black (*b* = −0.29, *p* < 0.001), Hispanic (*b* = −0.19, *p* < 0.001), and other race adolescents (*b* = −0.04, *p* = 0.03) exhibited fewer internalizing behaviors than did White adolescents. Adolescents living with poor families were more likely to exhibit internalizing behaviors (*b* = 0.06, *p* = 0.03). However, adolescents’ gender, age, mothers’ age, and mothers’ educational attainment were not associated with adolescents’ internalizing behaviors. In terms of externalizing behaviors, female adolescents were less likely to exhibit externalizing behaviors than were male adolescents (*b* = −0.08, *p* = 0.00). Hispanic adolescents were less likely to exhibit externalizing behaviors than were White adolescents (*b* = −0.10, *p* = 0.02). Mothers’ age was negatively associated with adolescents’ externalizing behaviors (*b* = −0.11, *p* < 0.001). Adolescents living with poor families were more likely to exhibit externalizing behaviors (*b* = 0.08, *p* = 0.00). However, adolescents’ age, the dummy variables for the Black and other racial categories, and mothers’ educational attainment were not significantly associated with adolescents’ externalizing behaviors.

### Moderating effect of school connectedness

**Figure 4:**
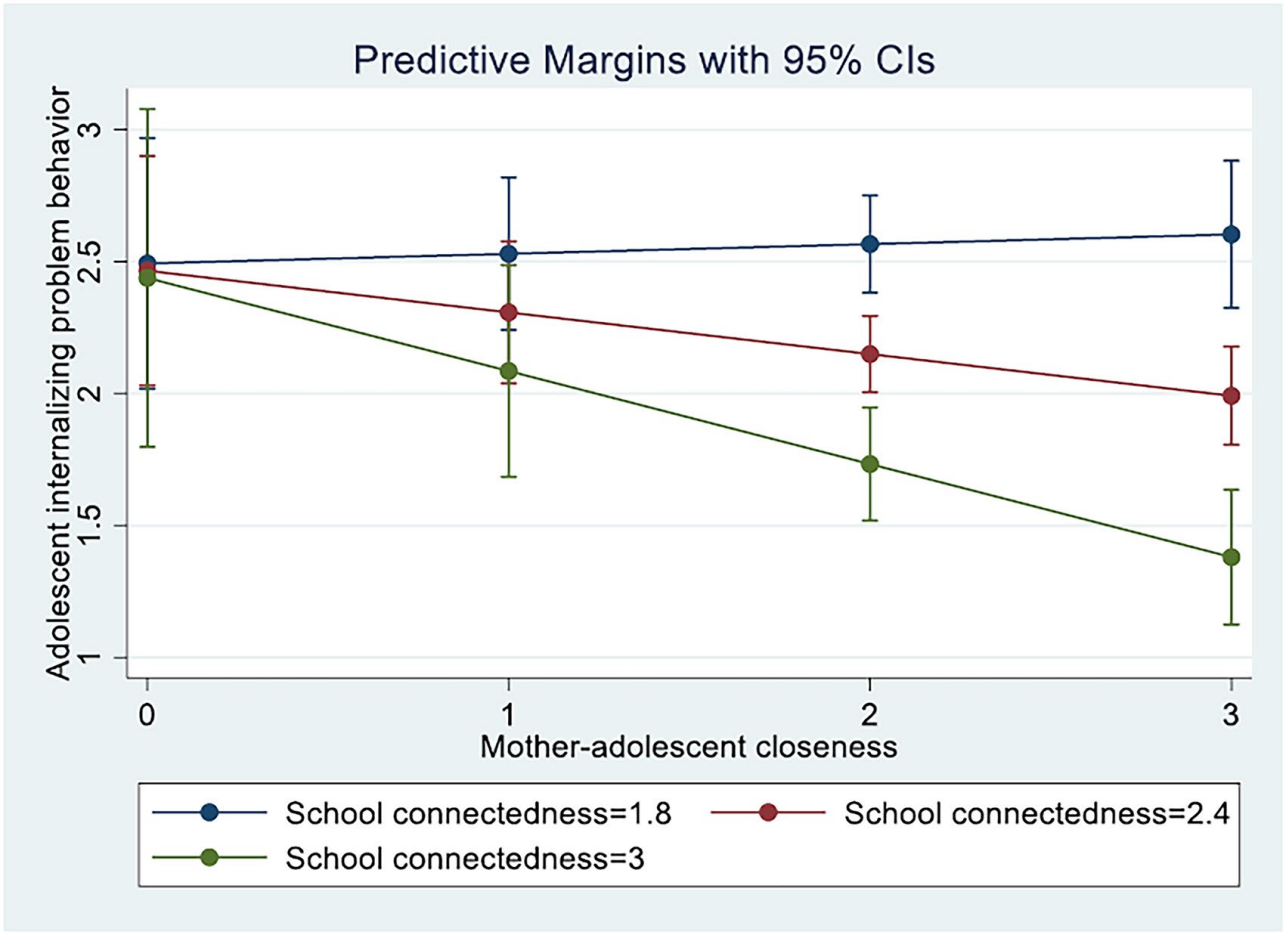
Interaction between school connectedness and mother-adolescent closeness in the prediction of adolescents’ internalizing behaviors.

Table 5 shows the results regarding the moderating role of school connectedness in the SEM model. Mother-adolescent closeness positively predicted adolescents’ internalizing behaviors (*b* = 0.62, *p* = 0.05), and this effect was moderated by school connectedness (*b* = −0.32, *p* = 0.01). In other words, the association between mother-adolescent closeness and adolescents’ internalizing problem behavior was weaker for adolescents with high levels of school connectedness. Figure 4 also shows that school connectedness moderated the association between mother-adolescent closeness and adolescents’ internalizing problem behaviors. These findings suggest that mother-adolescent closeness effectively reduces adolescents’ internalizing behaviors when levels of school connectedness are high.

**Table 5:** Moderating effect of school connectedness (*N* = 1,384).

Predictors	Adolescents’ internalizing behaviors								Adolescents’ externalizing behaviors							
	*b*	(SE)	*p*	Beta	*b*	SE	*p*	Beta	*b*	SE	*p*	Beta	*b*	SE	*p*	Beta
Mother-adolescent closeness	0.62	(0.31)	0.05	0.19	−0.12	(0.09)	0.20	−0.04	0.00	(0.66)	1.00	0.00	−0.90	(0.19)	<0.001	−0.13
School connectedness	−0.05	(0.30)	0.88	−0.01	−0.72	(0.13)	<0.001	−0.18	−0.60	(0.64)	0.35	−0.07	−1.41	(0.28)	<0.001	−0.16
Closeness school connectedness	−0.32	(0.13)	0.01	−0.32					−0.40	(0.28)	0.15	−0.18				
Mothers’ depression	0.20	(0.03)	<0.001	0.20	0.22	(0.11)	0.04	0.22	0.36	(0.06)	<0.001	0.17	0.41	(0.23)	0.07	0.19
Mothers’ depression school connectedness					−0.01	(0.04)	0.84	−0.02					−0.02	(0.09)	0.80	−0.03
Covariates																
Adolescent is female	0.08	(0.14)	0.54	0.02	0.10	(0.14)	0.47	0.02	−0.95	(0.29)	0.00	−0.09	−0.93	(0.29)	0.00	−0.09
Adolescents’ age	−0.11	(0.09)	0.21	−0.03	−0.11	(0.09)	0.24	−0.03	−0.12	(0.19)	0.52	−0.02	−0.12	(0.19)	0.55	−0.02
Adolescents’ race																
Black	−1.53	(0.25)	<0.001	−0.29	−1.54	(0.25)	<0.001	−0.29	−0.24	(0.52)	0.65	−0.02	−0.26	(0.52)	0.62	−0.02
Hispanic	−1.11	(0.28)	<0.001	−0.17	−1.13	(0.28)	<0.001	−0.18	−1.15	(0.59)	0.05	−0.09	−1.19	(0.59)	0.04	−0.09
Other race	−0.65	(0.35)	0.06	−0.06	−0.66	(0.35)	0.06	−0.06	−0.63	(0.74)	0.40	−0.03	−0.63	(0.74)	0.39	−0.03
Mothers’ age	−0.01	(0.01)	0.37	−0.03	−0.01	(0.01)	0.37	−0.03	−0.10	(0.02)	<0.001	−0.11	−0.10	(0.02)	<0.001	−0.11
Mother graduated from college	0.17	(0.21)	0.42	0.02	0.17	(0.21)	0.42	0.02	0.05	(0.44)	0.91	0.00	0.05	(0.44)	0.91	0.00
Family in poverty	0.19	(0.14)	0.19	0.04	0.19	(0.14)	0.19	0.04	0.91	(0.30)	0.00	0.09	0.91	(0.30)	0.00	0.09
Intercept	5.61	(1.62)	0.00		7.02	(1.52)	<0.001		14.45	(3.41)	<0.001		16.12	(3.20)	<0.001	
*F*(*df*)	14.02(12)		<0.001		13.45(12)		<0.001		15.20(12)		<0.001		15.01(12)		<0.001	
Adjusted *R*^2^	0.1137				0.1094				0.1228				0.1214			

Mothers’ depression positively predicted adolescents’ internalizing behaviors (*b* = 0.22, *p* = 0.04), but this effect was not moderated by school connectedness. Additionally, school connectedness did not significantly moderate the relationship between mother-adolescent closeness and adolescents’ externalizing behaviors or that between mothers’ depression and adolescents’ externalizing behaviors.

## Discussion

Although many studies indicate that adolescents in single-parent households are at heightened risk for problem behaviors, few studies have explored family processes leading to adolescents’ internalizing and externalizing behaviors. Applying the FSM, this study examined the processes in single-mother families that link material hardship to adolescents’ problem behaviors—namely, single mothers’ depression and mother-adolescent closeness. Specifically, with little research examining moderators in the FSM (Masarik and Conger, 2017), this study explored the moderating roles of adolescent school connectedness in the paths that link mothers’ depression and mother-adolescent closeness to adolescents’ problem behaviors.

The results supported our first hypothesis, indicating the sequential mediating roles of mothers’ depression and mother-adolescent closeness in the relationship between material hardship and adolescents’ problem behaviors. This aligns with previous research demonstrating single mothers’ psychological functioning and parenting as important sequential mediators in the relationship between family risk factors and African American adolescents’ adjustment (Kim and Brody, 2005). It is also similar to the results from previous studies investigating adolescents raised in two-parent families or targeting adolescents and their parents regardless of parents’ gender or family structure (Benner and Kim, 2010; Landers-Potts et al., 2015; Diggs and Neppl, 2018). This study indicates that economic strain is a significant extrafamilial stressor that exacerbates single mothers’ mental health and relationships with adolescents, ultimately leading to both internalizing and externalizing problems. Although many studies have focused on adjustment of younger children in single-mother families to understand the impact of single mothers’ diverse stressors (Mistry et al., 2002; Scaramella et al., 2008), this study confirms that family is still an important contributor to adjustment during adolescence during which peers, friends, and other extrafamilial networks take on more important roles.

Specifically, mother-adolescent closeness played a significant partial mediating role in the relationship between mothers’ depression and adolescents’ problem behaviors. Research indicates that warm, close relationships with parents fulfill adolescents’ basic psychological needs and contribute to secure attachment, which lead to adolescents’ behavioral and emotional adjustment (Soenens et al., 2019). On the other hand, poor communication and negative parent–child relationships do not significantly change as children develop into adolescents, and even worse interactions occur over time in single-parent families (Loeber et al., 2000). Based on the FSM, this study showed that the mother-adolescent relationship in single-mother families is significantly indirectly associated with material hardship through mothers’ depression. Specifically, previous studies have reported less frequent interactions and higher negativity in parent-adolescent relationships among single-mother or single-parent families compared to two-parent families (Baer, 1999; Laursen, 2005). By applying FSM, this study showed how material hardship and single mothers’ depression are associated with an important facet of the mother-adolescent relationship, which eventually led to problem behaviors. This result also supported the second hypothesis positing the role of mothers’ depression as a single mediator between material hardship and adolescents’ problem behaviors. As previous studies suggest, although parenting and the parent–child relationship are the primary mechanisms that explain the relationship between parents’ and children’s mental health, other elements such as emotion contagion and genetic vulnerability may also be important (Goodman and Gotlib, 1999; Goodman et al., 2020). Additionally, because this study focused on mother-adolescent closeness, potentially significant mediating roles of other facets of parenting, such as monitoring and autonomy granting, should also be considered in further research (Soenens et al., 2019).

Taken together, this study highlights the importance of interventions that address the deleterious effects of economic strain and depression in single-mother families. Above all, although parents’ extrafamilial stress (e.g., economic hardship) is an important factor that explains their children’s behavioral problems (Östberg and Hagekull, 2013), as Crnic and Coburn (2019) note, only a few interventions have been developed to address such material hardship. Additionally, most intervention programs for single mothers have focused on parents of younger children (e.g., Brown and Bhavnagri, 1996; Lipman and Boyle, 2005), notwithstanding many single mothers’ challenges in providing financial and educational support for adolescent children and difficulties in dealing with new parenting roles required for raising adolescents (Elliott et al., 2015; Lee et al., 2019).

Moreover, this study examined the moderating role of school connectedness in the associations of mother-adolescent closeness and mothers’ depression with adolescents’ problem behaviors. As expected, we found that school connectedness significantly moderated the relationship between mother-adolescent closeness and adolescents’ internalizing behaviors. Although little attention has been paid to this moderating variable, several studies have reported a significant association between school connectedness and adolescents’ problem behaviors (Gonzales et al., 2014; Marraccini and Brier, 2017; Olivier et al., 2020), aligning with our study findings. Specifically, considering that experiencing connection with others is an important psychological need linked to adolescents’ well-being (Ryan and Deci, 2000), it is likely that adolescents’ feelings of connectedness with friends, teachers, and school itself buffered the negative effect of distance from mothers on internalizing problem behaviors. Research has indicated that school can play an important role in nurturing resilience, the capacity to successfully adapt to challenges that threaten normal functioning or healthy development (Masten and Cicchetti, 2016; Masten and Barnes, 2018). Particularly, as this study found that school can protect against internalizing behaviors when adolescents in single-mother households experience negative family processes that originate from material hardship, enhancing school connectedness, which can provide multiple relationship opportunities for adolescents, should be highlighted in reducing internalizing behaviors.

However, unexpectedly, school connectedness moderated neither the relationship between mothers’ depression and adolescent internalizing and externalizing problem behaviors nor that between mother-adolescent closeness and adolescents’ externalizing behaviors. Because few studies have explored school connectedness’s moderating role in the FSM, we cautiously suggest a few possible explanations of our results. Above all, regarding the lack of significant moderation of school connectedness in the relationship between mothers’ depression and adolescents’ problem behaviors, the diverse pathways through which the former can affect the latter offer one explanation. That is, school connectedness may have a significant indirect effect on adolescents’ problem behaviors through aspects of familial relationships (as found in this study), rather than there being a direct association between mothers’ depression and adolescents’ problem behaviors, which can be explained by various potential mechanisms such as emotional contagion, genetic vulnerability, and ineffective discipline (Goodman and Gotlib, 1999). Additionally, this study produced inconsistent results on adolescents’ internalizing and externalizing behaviors in terms of the interactive effect of mother-adolescent closeness and school connectedness. A large body of research indicates co-occurrence and co-development of internalizing and externalizing behaviors. However, some studies also indicate that the antecedents and contexts of the two problem behaviors can differ. For example, depending on adolescents’ peer networks and school activity types, more engagement in school social networks can be associated with increased delinquency (Eccles and Barber, 1999). Despite the direct and significant association between mothers’ depression and adolescents’ internalizing problem behaviors, this study found no moderating effect of school connectedness in the association between mothers’ depression and adolescents’ internalizing problem behaviors. It is possible that school connectedness’s moderating effect on adolescents’ internalizing problem behaviors was offset due to the strong association between mothers’ depression and adolescents’ internalizing problem behaviors. Several studies have reported mothers’ depression as one of the strongest predictors of adolescents’ internalizing problem behaviors (Weissman et al., 1997; Goodman and Gotlib, 1999; Wolford et al., 2019). As school connectedness itself was significantly associated with adolescents’ internalizing problem behaviors, it may be possible that the influence of mothers’ depression offset this moderating effect.

Similarly, our findings contrast with previous evidence on the protective effects of school connectedness on adolescents’ problem behaviors. Because the adolescents of our sample were approximately 15 years of age, prospective studies need to explore whether the protective role of school connectedness is more evident among older adolescents. As previous studies have found stronger association between school connectedness and externalizing problem behaviors for older high school students (Resnick et al., 1997; Crosnoe et al., 2002), future studies should focus on later adolescents and examine whether school connectedness mitigates externalizing problem behaviors.

### Limitations

Although this study has many strengths, a few limitations must be noted. First, although this study used both mother-reported and teen-reported measures, there is a methodological limitation due to the use of a mono-method self-reported design, which may have led to inflated statistical associations and biased estimates. Future studies may consider including observational methods to measure constructs of interest, such as parent-adolescent closeness. Second, based on the FSM, this study investigated material hardship as an important stressor that may directly or indirectly affect mothers’ mental health, mothers’ relationships with their adolescent children, and adolescents’ problem behaviors. However, future studies should also consider diverse known risk factors in single-mother households that may influence family processes. That is, single mothers’ challenges should be addressed comprehensively to understand the complex family processes that explain adolescents’ problem behaviors (Weinraub and Kaufman, 2019). For example, insufficient social support, lack of coparenting, and adolescents’ childhood stressors and chronic problem behaviors could majorly affect family processes and adolescents’ life outcomes (Cairney et al., 2003; Sterrett et al., 2009; Daryanani et al., 2017). Third, this study’s cross-sectional design precludes making causal inferences. Although the directionality of our research model was guided theoretically by the FSM, the increasing effect of children on their parents during adolescence should also be considered. For example, the influence of adolescents’ problem behaviors on mothers’ depression and mother-adolescent relationships could be examined in the future. Additionally, as adolescents’ problem behaviors may have been influenced by their childhood development, future research may consider using a longitudinal design to clarify the directionality and causality of the complex mechanisms. Lastly, although our study focused on single-mother families, further research should consider diverse and complex family structures, including single-father families, and how their family processes influence adolescents’ healthy development.

### Conclusion

Despite some limitations, this study contributes to the literature as one of the few studies examining specific family processes in single-mother families to explain adolescents’ problem behaviors. Specifically, previous studies have indicated that adolescents in single-parent households are at a heightened risk for adjustment problems (Barrett and Turner, 2005; Amato, 2010; Daryanani et al., 2016). Although many studies suggest various risk factors for adolescent problem behaviors, investigating the processes associated with the behaviors is necessary (Childs et al., 2020). Specifically, although not all single parents live in poverty, single mothers are more likely to suffer from economic hardship compared to single fathers (United States Census Bureau, 2022). Accordingly, using samples in which households under the poverty threshold are overrepresented, this study revealed the significant indirect effect of material hardship on adolescents’ problem behaviors through mothers’ depression and the quality of mothers’ relationships with adolescents, which was guided by the FSM. Furthermore, with few studies investigating moderating effects in the FSM (Masarik and Conger, 2017), this study investigated the moderating role of school connectedness in the paths leading to problem behaviors. Although the moderating effect of school connectedness was significant only in the relationship between mother-adolescent closeness and internalizing problem behaviors, this study provides valuable initial findings regarding protective/risk factors in adolescents’ environments within the family stress process. Research should continue to elucidate the risk/protective factors that ultimately determine adolescents’ developmental outcomes. Additionally, considering that single mothers’ depression and mother-adolescent closeness are significant elements that explain the relationship between single mothers’ material hardship and adolescent problem behaviors, interventions that address emotional and relational problems associated with economic hardship in single-mother families should be devised.

## Data availability statement

Publicly available datasets were analyzed in this study. This data can be found at: https://fragilefamilies.princeton.edu/.

## Ethics statement

Ethical review and approval was not required for the study on human participants in accordance with the local legislation and institutional requirements. Written informed consent to participate in this study was provided by the participants’ legal guardian/next of kin.

## Author contributions

WL: conceptualization, methodology, writing — original draft, writing — review and editing, and supervision. YJ: software, formal analysis, data curation, writing — original draft, writing-review and editing, and project administration. All authors contributed to the article and approved the submitted version.

## Funding

This work was supported by the Ewha Womans University Research Grant of 2021.

## Conflict of interest

The authors declare that the research was conducted in the absence of any commercial or financial relationships that could be construed as a potential conflict of interest.

## Publisher’s note

All claims expressed in this article are solely those of the authors and do not necessarily represent those of their affiliated organizations, or those of the publisher, the editors and the reviewers. Any product that may be evaluated in this article, or claim that may be made by its manufacturer, is not guaranteed or endorsed by the publisher.
