# Supplementary material for: Examining family processes linked to adolescent problem behaviors in single-mother families: The moderating role of school connectedness
Source: Front Psychol. 2022 Sep 26;13:937698. doi: 10.3389/fpsyg.2022.937698 (PMC9549365; doi:10.3389/fpsyg.2022.937698)
Supplement: Supplementary file 1 [file Data_Sheet_1.pdf]

Supplementary Materials

Appendix 1. Supplementary analysis changing the path between mother-adolescent closeness and mother's depression

Table A1. SEM standardized coefficient estimates *(N = 1,384)*

Predictors	Direct effect							
	Mother-adolescent closeness		Mothers' depression		Adolescents' internalizing behaviors		Adolescents' externalizing behaviors	
	b(SE)	*p*	*b*(SE)	*p*	*b*(SE)	*p*	*b*(SE)	*p*
Material hardship	-0.08(0.03)	0.00	-		-		-	
Mother-adolescent closeness	-		-0.07(0.03)	0.01	-0.08(0.03)	0.00	-0.16(0.03)	<.001
Mothers' depression	-		-		0.20(0.03)	<.001	0.17(0.03)	<.001
Covariates								
Adolescent is female	-0.06(0.03)	0.05	-0.05(0.03)	0.06	0.03(0.03)	0.24	-0.08(0.03)	0.00
Adolescents' age	0.01(0.03)	0.69	0.01(0.03)	0.84	-0.02(0.03)	0.42	-0.01(0.03)	0.60
Adolescents' race (White = 0)								
Black	0.06(0.05)	0.20	-0.15(0.05)	0.00	-0.29(0.05)	<.001	-0.02(0.05)	0.61
Hispanic	0.06(0.04)	0.17	-0.17(0.04)	<.001	-0.19(0.04)	<.001	-0.10(0.04)	0.02
Other race	-0.02(0.04)	0.66	-0.07(0.03)	0.04	-0.07(0.03)	0.03	-0.04(0.03)	0.21
Mothers' age	0.01(0.03)	0.74	-0.04(0.03)	0.17	-0.02(0.03)	0.35	-0.11(0.03)	<.001
Mother graduated from college	-0.04(0.03)	0.13	-0.04(0.03)	0.19	0.00(0.03)	0.94	-0.02(0.03)	0.41
Family in poverty	0.03(0.03)	0.31	0.15(0.03)	<.001	0.06(0.03)	0.03	0.08(0.03)	0.00
Intercept	2.64(0.60)	<.001	1.13(0.58)	0.05	1.97(0.57)	0.00	2.43(0.58)	<.001

Figure A1. SEM path model from material hardship to adolescent internalizing behaviors

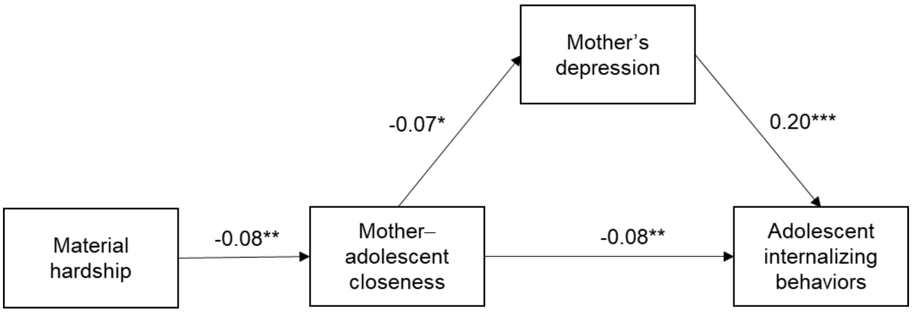

* $p < .05$, ** $p < .01$, *** $p < .001$

Figure A2. SEM path model from material hardship to adolescent externalizing behaviors

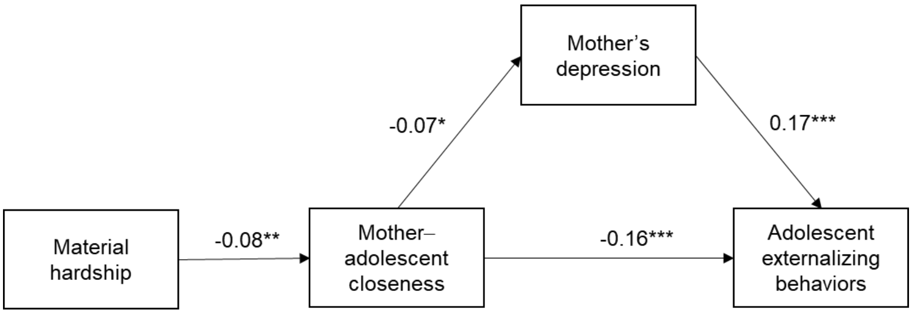

* $p < .05$, ** $p < .01$, *** $p < .001$

Table A2. Model Comparison

	Comparison	Log-likelihood	Df	AIC	BIC
Internalizing	Original Model	-19762.08	88	39700.16	40160.65
	Switched Model	-19822.07	88	39820.14	40280.62
Externalizing	Original Model	-20809.52	88	41795.04	42255.52
	Switched Model	-20869.67	88	41915.34	42375.82

Appendix 2. Supplementary analysis including both adolescent internalizing and externalizing behaviors

Figure A3. SEM path model including both adolescent internalizing and externalizing behaviors

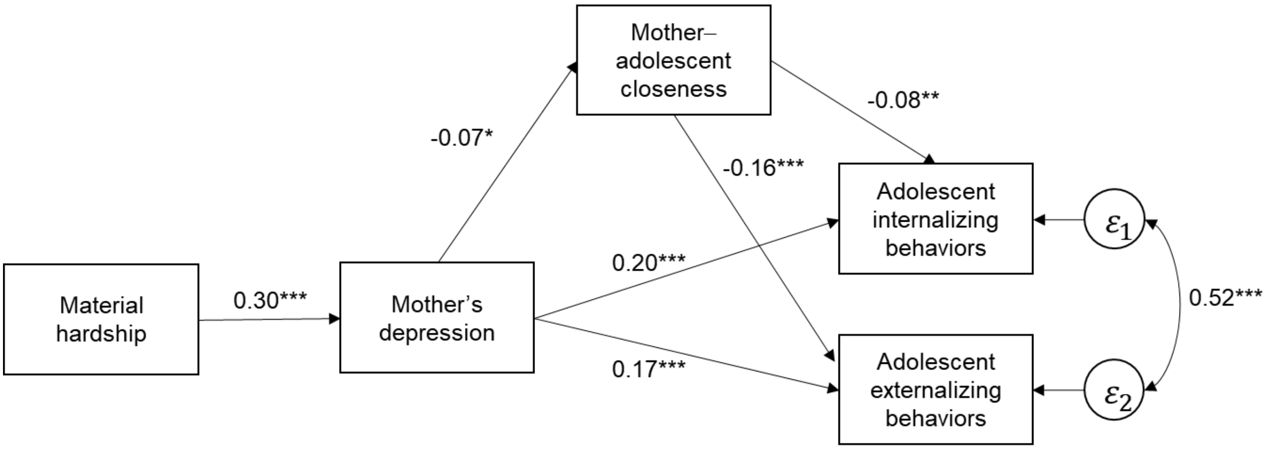

* $p < .05$, ** $p < .01$, *** $p < .001$; Error terms except adolescent internalizing and externalizing behaviors were excluded from the SEM diagram.

Table A3. SEM standardized coefficient estimates including both adolescent internalizing and externalizing behaviors

Predictors	Direct effect											
	Mother's depression			Mother-adolescent closeness			Adolescent internalizing behaviors			Adolescents externalizing behaviors		
	b	*SE*	p	b	*SE*	p	b	*SE*	p	b	*SE*	p
Material hardship	0.3019	*0.0249*	<.001	-			-			-		
Mother's depression	-			-0.0687	*0.0277*	0.013	0.2001	*0.0258*	<.001	0.1699	*0.0258*	<.001
Mother-adolescent closeness	-			-			-0.0750	*0.0262*	0.004	-0.1621	*0.0266*	<.001
Covariates												
Female	-0.0494	*0.0253*	0.051	-0.0585	*0.0275*	0.033	0.0304	*0.0259*	0.240	-0.0830	*0.0256*	0.001
Child's age	0.0090	*0.0260*	0.728	0.0127	*0.0276*	0.645	-0.0210	*0.0264*	0.424	-0.0141	*0.0269*	0.600
Child's race (White=0)												
Black	-0.1597	*0.0444*	<.001	0.0491	*0.0478*	0.304	-0.2842	*0.0451*	<.001	-0.0240	*0.0465*	0.711
Hispanic	-0.1450	*0.0415*	<.001	0.0575	*0.0447*	0.198	-0.1868	*0.0425*	<.001	-0.0999	*0.0434*	0.026
Other race	-0.0785	*0.0330*	0.017	-0.0232	*0.0354*	0.510	-0.0739	*0.0337*	0.029	-0.0427	*0.0344*	0.214
Mother's age	-0.0127	*0.0261*	0.628	0.0147	*0.0282*	0.602	-0.0249	*0.0265*	0.346	-0.1067	*0.0262*	<.001
Mother graduated from college	-0.0128	*0.0268*	0.630	-0.0406	*0.0289*	0.161	0.0022	*0.0274*	0.925	-0.0225	*0.0271*	0.416
Family in poverty	0.1015	*0.0263*	<.001	0.0266	*0.0288*	0.355	0.0587	*0.0269*	0.029	0.0782	*0.0267*	0.003
Intercept	0.4700	*0.5578*	0.399	2.5514	*0.5981*	<.001	1.9670	*0.5693*	0.001	2.4081	*0.5782*	<.001

Table A4. Original model (presented in Table 3 in the manuscript)

Predictors	Direct effect											
	Mother's depression			Mother-adolescent closeness			Adolescent internalizing behaviors			Adolescents externalizing behaviors		
	b	*SE*	p	b	*SE*	p	b	*SE*	p	b	*SE*	p
Material hardship	0.3019	*0.0249*	<.001	-			-			-		
Mother's depression	-			-0.0687	*0.0277*	0.013	0.2001	*0.0258*	<.001	0.1699	*0.0258*	<.001
Mother-adolescent closeness	-			-			-0.0750	*0.0263*	0.004	-0.1621	*0.0266*	<.001
Covariates												
Female	-0.0494	*0.0253*	0.051	-0.0585	*0.0275*	0.033	0.0304	*0.0259*	0.240	-0.0830	*0.0256*	0.001
Child's age	0.0090	*0.0260*	0.728	0.0127	*0.0276*	0.645	-0.0210	*0.0264*	0.424	-0.0141	*0.0269*	0.600
Child's race (White=0)												
Black	-0.1597	*0.0444*	<.001	0.0491	*0.0478*	0.304	-0.2853	*0.0451*	<.001	-0.0240	*0.0466*	0.607
Hispanic	-0.1450	*0.0415*	<.001	0.0575	*0.0447*	0.198	-0.1868	*0.0425*	<.001	-0.0999	*0.0434*	0.021
Other race	-0.0785	*0.0330*	0.017	-0.0232	*0.0353*	0.510	-0.0739	*0.0337*	0.029	-0.0427	*0.0344*	0.214
Mother's age	-0.0127	*0.0261*	0.628	0.0147	*0.0282*	0.602	-0.0249	*0.0265*	0.346	-0.1067	*0.0262*	<.001
Mother graduated from college	-0.0128	*0.0268*	0.630	-0.0406	*0.0290*	0.161	0.0022	*0.0274*	0.936	-0.0225	*0.0271*	0.408
Family in poverty	0.1015	*0.0263*	<.001	0.0266	*0.0288*	0.355	0.0587	*0.0269*	0.029	0.0782	*0.0267*	0.003
Intercept	0.4700	*0.5578*	0.399	2.5658	*0.5977*	<.001	1.9670	*0.5692*	0.001	2.4280	*0.5783*	<.001

Table A5. Model Comparison

Comparison		Log-likelihood	Df	AIC	BIC
Including Both Internalizing and Externalizing		-23770.48	101	47742.96	48271.46
Original Model	Internalizing	-19762.08	88	39700.16	40160.65
	Externalizing	-20809.52	88	41795.04	42255.52
